# Necrosis of the Os Calcis

**Published:** 1892-12

**Authors:** G. W. Sherbino

**Affiliations:** Abilene, Texas


### NECROSIS OF THE OS CALCIS.

G. W. Sherbino, M. D., Abilene, Texas.

My little boy, æt. twelve, was away from home on a visit, and one day he was taken with a pain in the heel of his left foot, at the end. This pain he described as though something were sticking there, with throbbing pain and tenderness swelling about the heel and the malleolus, the internal and external; this pain kept on increasing, day by day, for about two weeks, when I opened heel, and he seemed somewhat easier, but up to this time I was up with him night and day. He received remedies that seemed to be indicated, but none of them controlled his condition until he got white around the mouth with restlessness and picking at his nose. I gave him a dose of Cina. At this time it seemed as if he would go into spasms, but this remedy controlled the symptoms till I made another incision.

From this time on he received Calc-carb., Hepar-sul., Lyc., Silic., Sanicula, Rhus-tox., Sulph., Psorin.

He would go sometimes for two months without any remedy, but when he had any symptoms at all I would try to prescribe for him. I could see no benefit from any remedy but Sanicula and Psorin. They were given in the highest potencies. He had psora when a little child, and as I was away from home, and my wife was going to be at the homes of friends, she could not think of taking him there without trying to dry it up, so she used the common remedy, Sulphur and Lard. This is no doubt the cause of this constitutional trouble coming in the form of ostitis with necrosis. He was on crutches for one year, with constant discharge from the opening.

One of the leading allopaths in town recommended cutting down to the bone and taking out the pieces and scraping it, as it would never get well till it was done, but I told him that I thought it was a case to be treated constitutionally, and that he had this necrosed bone because he was sick. The sponginess gradually left, and the heel seemed to get into a more healthy condition, and gradually the tissues grew fast to the heel, and the bone was by this means forced out by nature or by the area becoming less and less, till the last piece was taken out, and now, over a year and a half, he has had no trouble or inconvenience from it, and is as sound as the well one. All of the neighbors thought I was trifling with him by not having him treated by surgery.

### Discussion.

Dr. Long—I wish to ask a question, which is suggested by that paper: Whether it is the custom of physicians of this Association to cut open or lance abscesses?

Dr. I. Dever—If you want to know whether it is advisable to lance an abscess or not, get one yourself and try it. I had an abscess about two years ago and could not sleep for the pain, and so I thought I would open it. I did so, but in two minutes I saw I had made a mistake. It hurt worse, and I believe it took longer to heal.

Dr. Hitchcock—I should like to call attention to a curious result of an accident upon myself. Four years ago I cut my thumb to the bone. I fastened the wound together with court plaster and it healed in a few days. But a short time afterward a small round swelling appeared near the corner of the nail, which remained about two years, causing no inconvenience. Then there was a discharge of a thick, white, cheesy substance near the root of the nail at the base of the swelling, which continued for two or three months. Later a white point appeared on the top of the swelling, and when opened more of the cheesy matter appeared. By cutting deep down into the opening thus made, the cause of the trouble was found to be a portion of the thumb-nail growing from a part of the matrix of the nail which had been severed by the original cut, and the result is a small nail is growing directly through the flesh about a quarter of an inch from the root of the natural nail.

Dr. I. Dever—Dr. Hering claimed that nature was blind, and as an illustration of the fact he showed us a lizard with two tails. The lizard's tail had been wounded, so that its tail was partly cut off, and as a result the lizard grew another tail out of the wound. This illustrates the same fact as Dr. Hitchcock's thumb-nail.

Dr. J. V. Allen—I think the lancing of abscesses depends upon circumstances. I had a boy of fifteen who had a perineal abscess. I prescribed for him the best I could. Rapid suppuration went on and the pus settled down in the left iliac region. There was no pointing. The patient was in a bad way. There was great prostration, cold sweats, and general collapse. I punctured the abscess, and the most offensive pus I ever smelled ran out in great quantity. There was immediate relief, and the patient recovered.

Dr. S. Long—Some eight or nine years ago THE HOMŒOPATHIC PHYSICIAN gave a very clear and decided article from the pen of Dr. C. C. Smith, giving a number of good reasons why an abscess should neither be lanced nor poulticed. The same applied to felons and furuncles. The article was so conclusive to me that I tried it on the next case I had, and the next case I had, strange to say, was myself. Like Dr. Dever, I walked the floor for two nights, and then I concluded that if it was not better by morning I would lance it. The following morning I found a perfectly loose core about the size of a marrow-fat pea. I think I made a mistake in removing the core, for it took three weeks to fill up the place. Since that I have not lanced nor have I poulticed, believing with Dr. Smith that the abscess, or furuncle, is merely the external evidence of an internal condition.

Dr. Tompkins—I am reminded of a case of abscess in the abdomen, brought on by violently jumping the rope. It was a very painful affair. It became impossible for the girl to use her right leg, so that for a time I was in doubt whether she was not having hip disease. It proved to be an abdominal abscess. I called in counsel, and it was lanced in the region of the right groin. As long as I continued to press out all the pus, the secretion increased and there was doubt whether the patient were going to recover. Of course I was medicating to the best of my ability. I concluded that I would allow the pus to fill the cavity and preserve it a chance to run out only as it should overflow. The cavity filled up and the child recovered, the improvement following so promptly on the adoption of this plan that it formed a striking hint that the daily excavation of the pus was a mistake.

Dr. Fincke—The abscesses that I have treated with remedies without lancing have healed without a cicatrix; but if they were poulticed, they were more apt to leave a cicatrix. Of all the cases that came to my hands, there was not one in which blood poisoning occurred. The high potency seems to prevent it. Nobody need be afraid to trust the indicated remedy. We have many fearful cases cured with the homœopathic remedy in small doses. I published one case of viper-bite by Dr. Thorer, that was cured with three doses of a few pellets of Lachesis$^{30}$. The woman had fever, the limb was swollen and bluish, the abdomen was swollen and the patient almost collapsed. The moral is that we must trust our medicines and give them a fair trial before resorting to chemical and mechanical expedients.

Dr. Baylies—It is hardly necessary to affirm that when an abscess is fully matured and shows the significant yellow point, that it may be prevented from forming an unsightly cicatrix by puncture. A recent case was that of a woman who had, I was told, a premonitory swelling under the left maxilla. Allopathic physicians applied an ointment and in a few hours the whole of the left side of the face and neck, as far down as the clavicle, was swollen red and hard, as if poisoned by the application; developing a phlegmonous erysipelas.

There was also œdema of the tongue, the sublingual tissues, and the fauces. Apis reduced the œdema of the mouth and throat, but the swelling and cellulitis increased and the patient became unable to separate the teeth.

Nocturnal aggravation, salivation, and involuntary deglutition strongly pointed out Mercurius; which was given with gradual amelioration. The inflammation became less extensive, and Hepar-sulphur was given for indications; an abscess matured and was *punctured* with a narrow bistoury over the left cheek. The swelling subsided in a day or two, and the abscess healed, leaving no visible cicatrix. This instance simply illustrates the occasional *need* of instrumental interference after sufficient homœopathic treatment.

Dr. Kimball—I had a case a year ago in which an attack of German measles was followed by much swelling under the angle of the jaw. It slowly improved, but there was pus there. She and her friends were very persistent that it should be lanced. I refused, and I afterward heard that it had been lanced and a large amount of pus evacuated. In about a year she came to see me. She was thin and pale, with a suspicious cough, and said she had never been well since the abscess was opened. Under the action of medicine she is slowly improving, but this is not an encouragement to me to lance abscesses.

Dr. Bell—In inflammatory affections like carbuncles, furuncles, and styes I believe it is far better to give the indicated remedy, for in such cases evacuation will give no relief whatever. But there are cases in which the pus is the chief element of danger, where it is liable to burrow under deep fascia, and have no tendency to come to the surface, and will thus do great damage. Only a short time ago we had a case in the hospital in which an abscess had formed deep in the neck on the right side. It was a very dangerous form of disease, known as post-pharyngeal abscess. The patient could not lie down, could hardly eat or drink, and of course could sleep but little. We etherized him with great difficulty, for he was in constant danger of suffocation. We cut rapidly down and opened an abscess. The relief was immediate. He was immediately able to eat, sleep, and drink.

This is where the judgment of the physician comes in. We should endeavor to discriminate, for there are cases where the pus burrowing in deep tissues constitutes a grave danger, and where skillful lancing is necessary. To prevent the open capillaries from absorbing anything, I often prefer to use the thermo-cautery, which sears and hermetically seals them up.

Dr. E. E. Case—I had a case about two weeks ago that may be of interest in this connection. A farmer in bottling cider received a cut on the finger from a broken bottle. He took sugar and closed the lips of the wound and went on with his work. In a creamery he put his hand in ice cold water very frequently. There was no trouble for a week. The hand, arm, and axillary glands then became swollen and painful. When he came under my care the whole hand was enormously swollen and he was having chills and fever. I put him under treatment and succeeded in arresting the pain and general swelling. The skin of the finger was tense and plainly full of pus. The skin was very thick and tough. I knew there was only a calloused skin between the pus and the air. What should I do? Would it harm the patient to evacuate the pus? I made a small opening in the skin with instant relief to the pain. He slept well during the following night, and has been improving ever since.

Dr. Winn—A machinist came to me with a septic wound in the knuckle, occasioned by scratching it against a piece of brass, dirty with grease. It pained him slightly for several days, then a fever came on. Temperature rose to 104.6; pulse, 140. He had a terrific backache; no urine for twenty-four hours; no sleep for two days; dry, coated tongue with red tip. He was in a very bad condition. I opened the abscess, letting the incision follow the course of the tendons, and put a cold compress on it. Internally I gave him Rhus-tox. Next morning the temperature had dropped and he began to pass urine. The succeeding day the temperature was normal, and he recovered. Some of my friends told me that it was the Rhus that did the whole thing, but I feel differently. The great restlessness, backache, and red-tipped tongue were the indications for the remedy.

Dr. Tompkins—In such a case, if the indications for the remedy were certain, I would trust it to relieve the entire condition; but if I were not sure of the remedy, I would use some other means of relieving the patient.

Dr. Hoyne—Early in May one of my patients complained of having taken cold. He said that it acted like fever and ague, and from his description I thought he was right. The next day he did not have any; the third day he had a regular paroxysm. Under Bryonia the paroxysm ceased—that is, on the next day he should have had one it did not appear. About this time there appeared a swelling on the arm with pain. It was a little larger than a hickory nut. It gradually increased until the whole arm was swollen, including the hand. There was copious sweat and a typhoid looking tongue. At this time I was called out of town, leaving the case with Dr. King. During my absence Dr. King gave him Bryonia, and when I returned I found it still indicated. The temperature was less and the pain less, but the swelling was as bad as ever. He refused to have it opened. The man died of pyæmia, as I believe. Possibly his life might have been saved if the pus had been let out. The entire time of the sickness did not exceed fifteen days.

Dr. Sawyer—I have had some bad cases of abscess recently. One near the right ovary and probably involving the vermiform appendix. I was very much afraid that the patient was going to die. I had no faith in the patient's being able to endure an operation, and knowing nothing better to do I stuck to the remedy, and to my surprise and pleasure the pus disap-

36

peared without surgical interference. The swelling was enormous.

Dr. J. V. Allen—The pus disappeared down the fallopian tube, as it generally does in these cases. I had such a case in which operation was advised, and promise of death held out if refused. I was called in and prescribed for her. I made about four visits. The medicine relieved her, the pus broke, passed through the fallopian tubes and out of the vagina.

Dr. Sawyer—I do not know that I mentioned the fact that the pus did *not* pass out of the vagina or rectum, as I supposed it would, and told the nurse I expected such to occur, and it was watched. No pus passed. It must have been absorbed.

Dr. J. B. G. Custis—I do not like to find fault with Dr. Allen, and I have no doubt about the pus getting out, as he says, but I do doubt very strongly his opinion as to the course it took. This is a very interesting subject, but we must be a little careful about it. We cannot have every case go out of our hands as we would like it to go. In Washington we had a great many cases of abscess following malarial fevers and the grippe last winter, and it has been our rule to lance them unless we had a hectic fever come in. The amount of pus is nothing, the swelling amounts to nothing unless you get that continuous hectic fever. Then the safest plan is to get that pus evacuated. The location of the abscess has much to do with it, for if there is an exit for the pus anywhere it may, if left alone, open in a very unfavorable way and make trouble. The safest rule is to be guided by the thermometer and the condition of the patient. This is especially the case in abscesses about the mastoid process, and the ears. There may be great swelling there without great damage, but if you get elevated temperature the sooner you let the pus out the better for the patient and for your own reputation.

Dr. Hastings—I am a living example of the utility of opening an abscess. Two years ago at this time it was a question whether I was to remain any longer on this earth. The abscess, which was of the kidney, was opened, and I was considerably better very soon. I had suffered with this abscess for two years, that is, there was discharge of pus through the pelvis and ureters into the bladder, and had been at intervals for two years. I was not much inconvenienced by the discharge of the pus itself, but my health began to fail. I was passing two ounces of pus daily. I was obliged to give up business entirely. There was considerable disturbance of the digestive organs, terrible retching followed the ingestion of food. I was under the best homœopathic treatment that Boston afforded, Drs. Wm. P. Wesselhœft and James B. Bell. Finally I went out of town to Dr. Wesselhœft's country home, but I grew worse; the pain became unbearable in the left groin. Both my days and my nights were sleepless. I ate absolutely nothing during the two weeks I was at this country place. I was taken home on a bed worse than when I went away. Two days after I decided for myself that I would undergo an operation. The time was set for Saturday, but Thursday night was such a night of suffering that I determined to have the operation the next day. It was performed with complete relief. A large quantity of pus was evacuated and in two days I was able to retain food. If that abscess had not been opened I would not be here to tell the story. I suffer no discomfort, although there is a small fistula with slight discharge of pus.

Dr. J. B. Bell—At the operation we called together the staff of the Massachusetts Homœopathic Hospital. The pus was not in the kidney, but in the capsule. If you could have seen Dr. Hastings at that time and now, it would be difficult to realize it was the same person.

Dr. S. Long—It is generally said that there is always an exception to a rule, and I can readily understand that we cannot make an absolute rule to govern every individual case. I can say that I am glad that we have surgeons and surgical operations to preserve life and restore health. I am glad to know that the patient endured two years' suffering before a surgical operation was undergone, but I do not believe that the law of similars failed in that case. The failure was on the part of the gentlemen to find the simillimum. We must not blame or put down against the law what is due to the inability of man.

Dr. R. L. Thurston—The mere fact that the *fistula* exists shows that the patient is not well yet. The psora finds a vent in this new condition, and I believe that if the fistula were now to be removed by means of the knife a worse condition would arise. The operation simply diverted the disease into a new channel. The attending physicians failed to cure with their remedies, but Homœopathy did not fail.

Dr. C. E. Hastings—Nobody says, that I know of, that Homœopathy failed in my case. I grant that the simillimum was not found. I believe that the simillimum exists that would heal this fistula, but in the present state of our knowledge it might be difficult to find. Perhaps it has not been proved yet. I should be very happy to submit my case to Dr. Thurston or to any one here who thinks he can cure it. This is the ground on which I stand: I believe fully in the law of similars, and when we have reached its height, depth, and breadth I know not what wonders it will accomplish, but until that day we are compelled, on rare occasions, and under protest, to resort to surgical measures in justice to ourselves and our patients.

Dr. McLaren—And after all has been said it still remains the truth, that surgery is the opprobrium of medicine.

Adjourned to 10 A. M.

